# Deep learning based on MRI for assessing the prognostic value of lateral lymph nodes in rectal cancer

**DOI:** 10.3389/fonc.2025.1681939

**Published:** 2025-11-11

**Authors:** Guanzhong Qiao, Lili Feng, Zhenhui Li, Qiong Wu, Yulin Liu, Jie Zhao, Hao Jiang, Ke Zhao, Yanfen Cui, Huijie Jiang

**Affiliations:** 1 Department of Radiology, The Second Affiliated Hospital of Harbin Medical University, Harbin, China; 2 Department of Radiology, Sun Yat-sen University Cancer Center, Guangzhou, China; 3 Department of Radiology, Yunnan Cancer Hospital, Kunming, China; 4 Department of Radiology, Sun Yat-sen University Sixth Affiliated Hospital, Guangzhou, China; 5 Department of Radiology, Guangdong Provincial People’s Hospital, Guangzhou, China; 6 Department of Radiology, Shanxi Cancer Hospital, Taiyuan, China

**Keywords:** rectal cancer, lateral lymph node, artificial intelligence, deep learning, MRI

## Abstract

**Objectives:**

Accurate preoperative evaluation of positive lateral lymph node (LLN) is crucial for optimizing treatment strategies in rectal cancer. Traditional methods, such as MRI T2-weighted imaging (T2WI), face limitations like interobserver variability and difficulty detecting small or occult metastases. Deep learning (DL) may provide a more efficient and precise alternative.

**Methods:**

In this multicenter, retrospective study, images from 1,000 patients across five centers were annotated to train a DL model for identifying and segmenting LLN. The model was tested on images from 480 patients in a validation cohort. Kaplan-Meier analysis compared disease-free survival (DFS) and overall survival (OS) between LLN-positive and LLN-negative groups, while Cox regression identified prognostic factors for DFS and OS.

**Results:**

The DL model achieved an accuracy of 87.5% and a specificity of 73.8% in predicting LLN positivity, demonstrating high diagnostic performance. Both univariate and multivariate Cox regression analyses identified LLN status, circumferential resection margin (CRM), and tumor downstaging (TD) as independent prognostic factors. Kaplan-Meier analysis showed patients with positive LLNs had worse outcomes, with 3-year DFS of 57.66% vs. 81.66%, and 5-year OS of 61.62% vs. 84.82% compared to LLN-negative patients.

**Conclusions:**

The DL model effectively predicts positive LLNs, offering an efficient alternative to traditional methods and supporting preoperative decision-making. Its clinical implementation could enhance risk stratification and personalize therapeutic strategies for rectal cancer patients.

## Introduction

Colorectal cancer ranks as the third most commonly diagnosed malignancy worldwide, with rising incidence rates in many regions despite advancements in screening and treatment (1). Lymph node metastasis is a key independent prognostic factor for patients with rectal cancer (2, 3). In 1895, Gerota first proposed that mid- to low-rectal cancers could metastasize to lateral lymph nodes (LLN), a theory later confirmed by anatomical and pathological studies (4, 5). LLN metastasis typically involves the internal iliac, external iliac, obturator, and other pelvic lymph nodes. Studies have shown that the rate of LLN metastasis in rectal cancer ranges from 10% to 20% (6). The presence of LLN metastasis often indicates locally advanced disease and is associated with an elevated risk of local recurrence and distant metastasis.

Extensive research has established LLN metastasis as an independent prognostic factor in rectal cancer. Japanese surgical guidelines strongly recommend performing lateral lymph node dissection (LLND) during rectal cancer resection, particularly for high-risk cases. The Japanese Society for Cancer of the Colon and Rectum (JSCCR) guidelines specify that LLND should be considered in patients with low-lying rectal cancer when preoperative imaging identifies enlarged LLNs (≥7 mm in short-axis diameter) (7, 8). The JCOG0212 trial demonstrated that combining total mesorectal excision (TME) with LLND achieved significantly lower rates of local recurrence (7.4% vs. 12.6%) and pelvic sidewall recurrence compared to TME alone (9). These findings suggest that LLND may reduce recurrence risk and enhance survival outcomes, especially in populations with advanced rectal cancer. Precise preoperative evaluation of LLN status is essential for tailoring treatment strategies, as it identifies patients who would benefit most from targeted lymphadenectomy. However, the consistent and accurate identification of these high-risk nodes remains a significant challenge in clinical practice, creating a critical need for more objective and automated diagnostic tools.

With advancements in artificial intelligence (AI), particularly in automated image analysis, deep learning (DL) techniques now enable precise segmentation by automatically learning image features (10–12). nnUNet is a medical image segmentation AI model based on U-Net that self-configures network architecture and training parameters through rule-based automation, enhancing accuracy and efficiency while adapting to diverse medical imaging tasks without manual intervention (13–15). Overall, DL offers an efficient, accurate, and automated solution for MRI-based evaluation of lymph node metastasis in rectal cancer, improving diagnostic accuracy, reducing clinician workload, and enhancing reliability.

This DL model automates the segmentation and detection of LLN metastasis in rectal cancer using T2WI, reducing interobserver variability and accelerating preoperative assessment. As LLN positivity is a key prognostic factor, the model aids in precise risk stratification and personalized therapeutic strategies, enhancing patient management and optimizing oncological outcomes.

## Methods

### Patient enrollment

Data were retrospectively collected from patients across three institutions—Shanxi Cancer Hospital (SXCH), Sun Yat-sen University Cancer Center (SYSUCC), and the Second Affiliated Hospital of Harbin Medical University (HMU2)—spanning from January 2013 to December 2023. The study cohort comprised 1,114 patients. Following exclusion of 114 cases (10.2%) for poor image quality or insufficient data, high-quality images from 1,000 patients were annotated, resulting in a curated dataset used to train the model. This cohort was used as the training set for manual annotation, data preprocessing, and constructing the DL model. A separate validation cohort comprising 480 patients—after excluding 15 cases (3.1%) due to poor image quality or incomplete data—was recruited from the Sixth Affiliated Hospital of Sun Yat-sen University (SYSU6) and Yunnan Cancer Hospital (YNCH) between January 2013 and December 2023. This cohort, consisting of high-quality images, was used to evaluate the performance of the DL model.

LLN positivity was determined by consensus MRI interpretation based on criteria including short-axis ≥ 7 mm or presence of at least two malignant features (irregular border and heterogeneous signal) (7, 8). Due to the complications caused by LLND, such as genitourinary dysfunction and intraoperative bleeding, there are challenges in obtaining informed consent from patients. Therefore, all cases included in this study without LLND.

The specific inclusion and exclusion criteria are shown in **Supplementary Method 1**. The recruitment process is shown in **Supplementary Figure S1**. Both cohorts included pre-treatment pelvic MRI T2WI and several clinical data **Supplementary Method 1**. The study complied with the Declaration of Helsinki and received approval from the Ethics Committee of the HMU2 (approval number: YJSKY2024-322).

The clinical data, including age, sex, histological grade, and baseline levels of carcinoembryonic antigen (CEA; normal range: 0–5 ng/mL), were extracted from the electronic medical records of each patient. Additionally, two radiologists with specialized expertise in colorectal cancer imaging evaluated other relevant clinical characteristics, such as the short-axis diameter, circumferential resection margin (CRM), MRI-detected extramural vascular invasion (mrEMVI), tumor size, and T stage, N stage. mrCRM positivity was defined as a tumor-to-mesenteric fascia or levator muscle distance of ≤ 1 mm. Pathological complete response (pCR) was defined as the complete absence of residual tumor cells in the surgical specimen following neoadjuvant treatment.

### MRI data acquisition and standardization

Axial high-resolution T2WI were acquired using 1.5 T or 3.0 T scanners, and the parameters for MRI scans are summarized in **Supplementary Table S1**. Raw MRI data were exported from the PACS in DICOM format and evaluated by radiologists using RadiAnt DICOM viewer. The radiologists assessed various indicators, including tumor location, mrN stage, mrT stage, mr-extramural vascular invasion (mrEMVI) status, mr tumor downstaging(mrTD), and LLN status. A 7 mm cutoff for the maximum short axis was applied for LLN delineation. LLNs smaller than 7 mm were considered positive if two or more malignant features were present (16, 17). Two radiologists with more than five years of experience conducted the assessments. Any discrepancies were resolved by a senior radiologist with over ten years of experience.

To mitigate heterogeneity arising from multi-institutional scanners and imaging protocols (2013–2023), all images underwent a standardized preprocessing pipeline. Spatial resolution was first unified through cropping and bilinear interpolation to a consistent matrix (18, 19). Subsequently, Z-score normalization was applied to standardize pixel intensity distributions across scanners. Finally, data augmentation techniques—including rotation, flipping, translation, scaling, and contrast adjustment—were employed to further enhance model robustness and generalizability (20–22).

### Development of a DL-based LLN segmentation model

The region of interest (ROI) was delineated using ITK-SNAP software (version 4.1.0) by two radiologists with 5 and 20 years of experience in colorectal and abdominal lesion diagnosis, respectively. Following the methodology of Ogura et al. (23), the radiologists independently annotated lateral lymph node (LLN) partitions on T2-weighted images (T2WI) from 1,000 training cases, using distinct color labels for positive and negative nodes. Discrepancies were resolved through consensus review. Established protocols were strictly adhered to throughout the modeling process (24). We utilized the first version of nnU-Net for both training and inference. While retaining the default full-resolution 3D pipeline, mirror-based data augmentation was excluded due to the anatomical asymmetry of the femoral heads, which could result in incorrect left-right label assignments. The model architecture followed the standard nnU-Net configuration, consisting of a five-level U-Net with 32 base filters, and employed a combined Dice and Cross-Entropy loss function in a 0.7:0.3 ratio.

A rigorous five-fold cross-validation scheme was employed, using non-overlapping 80:20 splits for training and validation in each fold. All training and inference procedures were executed on a workstation equipped with an Nvidia RTX 4090 GPU and AMD 7950X CPU. During inference, nnU-Net’s ensemble method was used to aggregate outputs from all five cross-validation models, thereby enhancing prediction robustness. Model performance was evaluated on the validation sets, with further optimization achieved by selecting the top-performing models.

### Evaluation of DL model performance

In the validation cohort, three radiologists manually delineated the regions of interest according to the same protocol. Two of the surgeons had 5 years of experience, while the validation was conducted by a certified radiologist with 10 years of experience in evaluating colorectal cancer imaging. Meanwhile, the surgeons independently assessed lymph node status blinded to the patient’s clinical or pathological information. The prediction results from the trained DL model were recorded as DLLLN+(Positive) or DLLLN−(Negative). Then, we used Cohen’s Kappa test to assess both the interrater agreement among radiologists and the agreement between the DL model’s predictions and each radiologist’s evaluation. This statistical measure helped us quantify the level of consensus beyond chance, providing a clear indication of the reliability of the radiologists’ interpretations and the accuracy of the DL model’s assessments.

### Statistical analysis

Continuous variables are presented as mean ± standard deviation (SD), and categorical variables as proportions or composition ratios. Student t-test or the Mann–Whitney test was used to analyze the intergroup comparisons of continuous variables. The χ² test was used to analyze the categorical variables. Kaplan–Meier curves were constructed to compare the 3, 5-year DFS and OS rates among the various groups. Univariate and multivariate Cox regression analyses were performed to identify independent prognostic factors for patients with rectal cancer. *p* < 0.05 was considered statistically significant. All statistical analyses were conducted using RStudio, SPSS and GraphPad. Variables with a p-value < 0.10 in the univariate analysis or those considered clinically relevant were included in the multivariate Cox regression model.

## Results

### Clinical characteristics

Between September 2009 and June 2020, a total of 1,480 individuals were enrolled in the study. The validation cohort comprised 480 patients selected from two independent research groups, with a mean age of 54.89 ± 14.43 years. Among these patients, 335 (69.8%) were male, and 145 (30.2%) were female. The majority had mid-to-low rectal cancer, with a median survival time of 25.4 months. The 3-year survival rate was 39%. For each patient in the validation cohort, the region of interest (ROI) was manually delineated on their T2WI scans and assessed by two radiologists by a radiology expert. Then, these patients were categorized into mrLLN+(positive) and mrLLN−(Negative) group. A total of 61 patients were diagnosed with positive LLNs according to the DL model.

### Segmentation performance of the DL model

**Figure 1:**
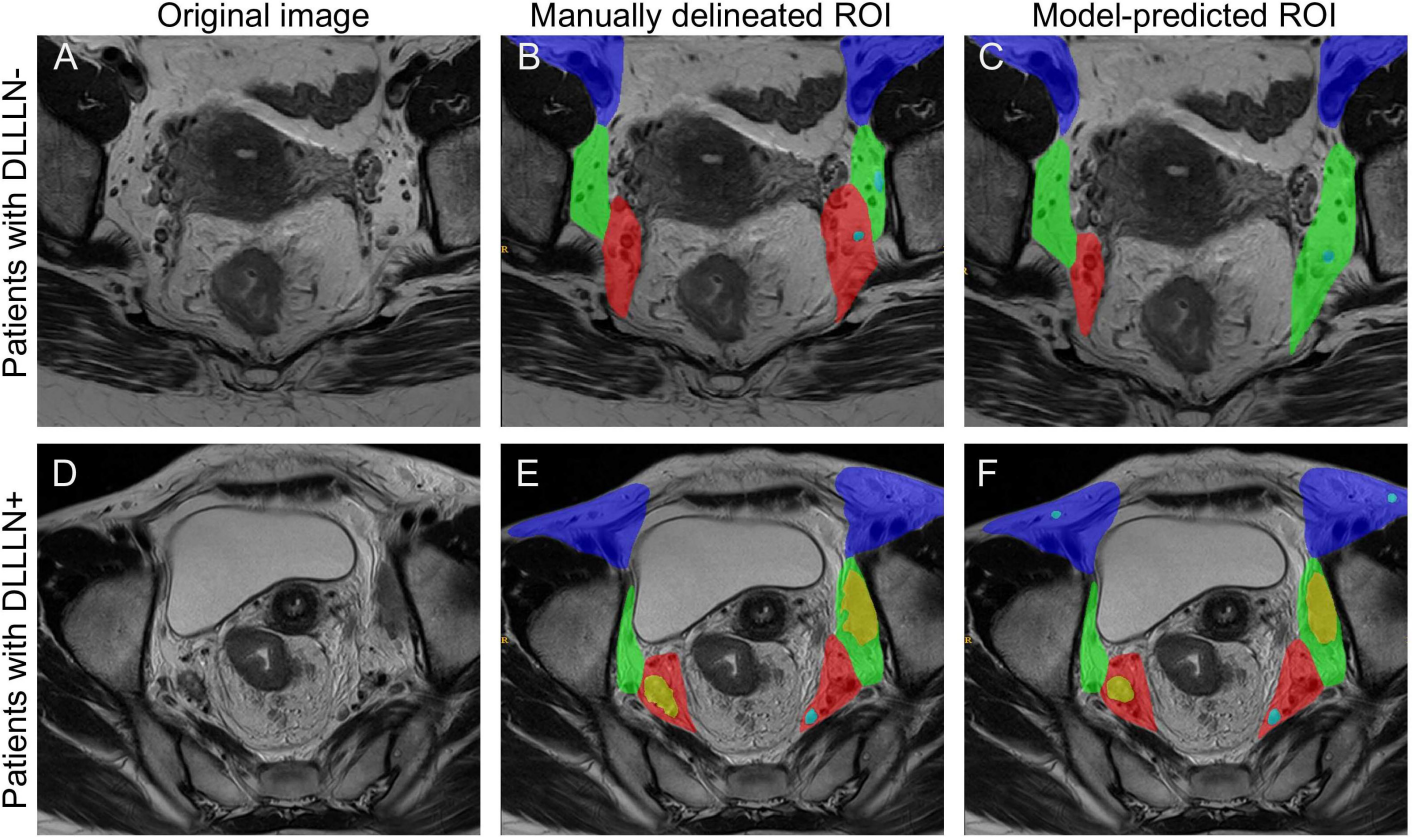
Comparison of the manually delineated regions of interest (ROI) and deep learning (DL) model predictions for the same case. **(A–C)** The original T2-weighted image, the manually delineated region of interest (ROI), and the model-predicted ROI for a LLN^-^ patient, respectively. **(D–F)** The corresponding images for another patient, including the original image, manually delineated ROI, and model-predicted ROI for a LLN^+^ patient. The color coding is as follows: the purple area indicates the external iliac and inguinal regions, the green area represents the internal obturator region, the red area corresponds to the internal iliac region, blue nodes denote negative lymph nodes, and yellow nodes indicate positive lymph nodes.

**Figure 1** presents the processed T2Ax images of two patients: one with positive lateral lymph nodes and one without. The trained model was used to predict positive LLNs in patients from the validation cohort. The results indicated that, among the 480 patients in the validation cohort, 90 were diagnosed with positive LLN(DLLLN+), while 390 were evaluated with negative LLN(DLLLN-). **Table 1** summarized the differences in baseline characteristics between patients with DLLLN+ and DLLLN- as identified by the DL model. We found that the distribution of mrN stage, mrEMVI, mrTD, pCR status showed significant differences between the two groups (*p* < 0.05).

**Table 1:** Descriptive analysis of patients in validation cohorts stratified by AI-LLN.

	DLLLN-	DLLLN+	*P*
Age			0.352
Mean ± SD	55.3 ± 11.2	52.6 ± 12.7	
Sex			0.836
Male	273 (70.0)	62 (68.9)	
Female	117 (30.0)	28 (31.1)	
CEA level			0.298
Normal	226 (58.2)	47 (52.2)	
Abnormal	162 (41.8)	43 (47.8)	
Location			0.064
Low	191 (49.0)	55 (61.1)	
Middle & High	199 (50.9)	35 (38.9)	
mrT stage			0.165
mrT1-T3	331 (84.9)	71 (78.9)	
mrT4	59 (15.1)	19 (21.1)	
mrN stage			<0.001
N0-N1	268 (68.7)	44 (48.9)	
N2	122 (31.3)	46 (51.1)	
mrEMVI status			0.003
Negative	288 (73.8)	52 (57.8)	
Positive	102 (26.2)	38 (42.2)	
mrTD status			0.002
Negative	319 (81.8)	70 (66.7)	
Positive	71 (18.2)	30 (33.3)	
mrCRM status			0.003
Negative	275 (70.5)	49 (54.4)	
Positive	115 (29.5)	41 (45.6)	

mr, magnetic resonance; DL, deep learning; CEA, carcinoembryonic antigen; EMVI, extramural vascular invasion; LLN, lateral lymph node; TD, tumor deposit; CRM, Circumferential Resection;

a The normal values for CEA level range from 0 to 5 ng/ml.

**Figure 2:**
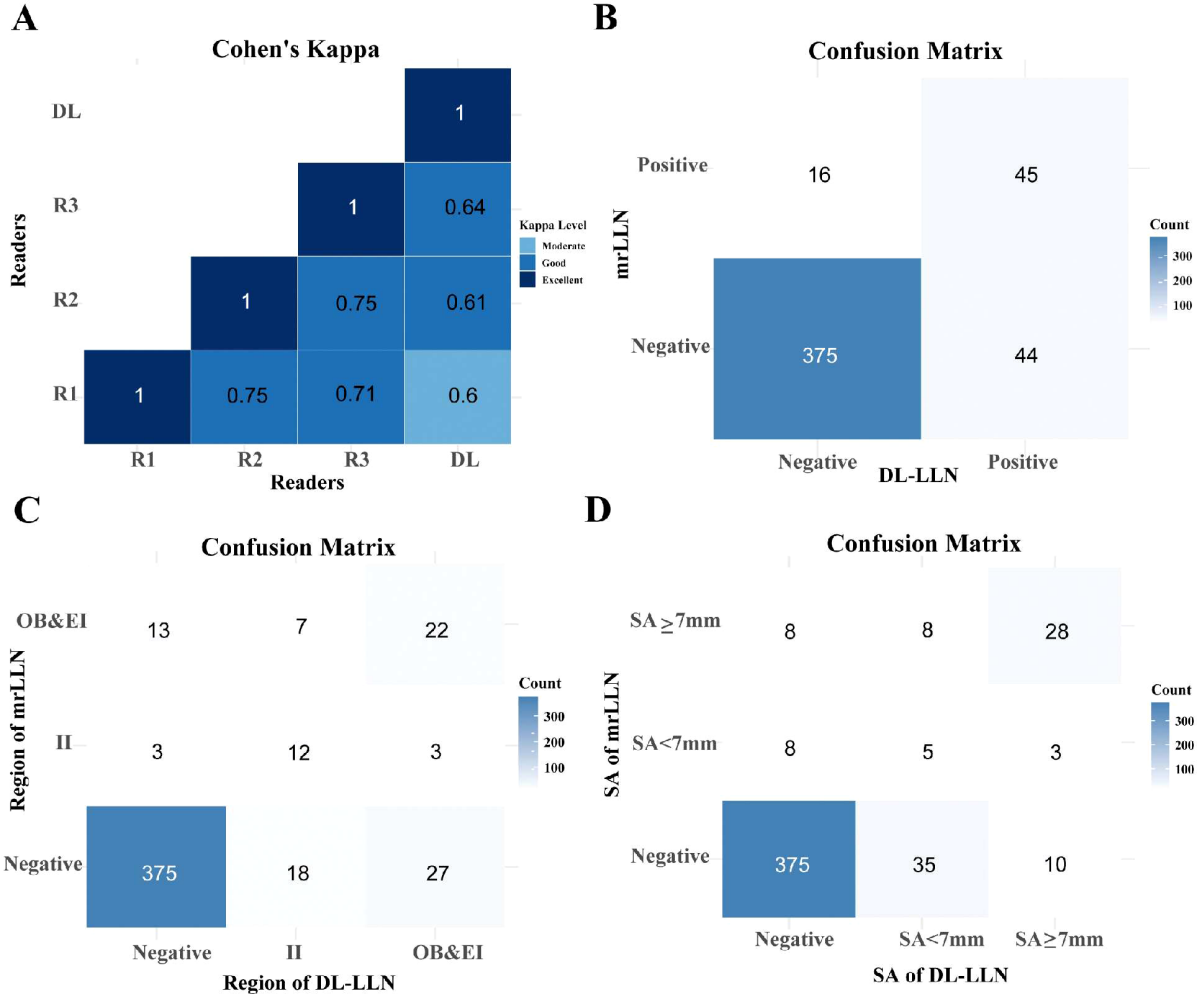
Examining the agreement between three radiologists and the AI model using Cohen’s Kappa. **(A)** Heatmap of Cohen’s Kappa showing the agreement between the junior radiologist (R1), mid-level radiologist (R2), senior radiologist (R3), and the deep learning (DL) model predictions. **(B)** Confusion matrix comparing the DL model’s predictions with the combined mrLLN assessment from all three radiologists. **(C)** Confusion matrix for the DL model’s prediction of lateral lymph node regions. **(D)** Confusion matrix for the DL model’s prediction of the maximum short-axis diameter of lateral lymph nodes. Abbreviations: R, reader; AI, artificial intelligence; LLN, lateral lymph node; mr, magnetic resonance; II, internal iliac; OB, obturator; EI, external iliac.

A Cohen’s Kappa analysis was conducted to evaluate inter-rater agreement among the radiologists (**Figure 2A**). To evaluate the consistency of diagnostic outcomes between the deep learning (DL) model and radiologists, we employed kappa statistical analysis. According to the criteria proposed by Landis and Koch (1977), kappa values are interpreted as follows: 0.00–0.20 (slight agreement), 0.21–0.40 (fair agreement), 0.41–0.60 (moderate agreement), 0.61–0.80 (substantial agreement), and 0.81–1.00 (almost perfect agreement). The DL model and the senior radiologist demonstrated a kappa value of 0.64, indicating clinically acceptable agreement comparable to expert variability. Ultimately, results unanimously agreed upon by all three radiologists were retained, with any discrepancies resolved through a consensus discussion. The DL model demonstrated high diagnostic performance in predicting LLN positivity, achieving an accuracy of 87.50% (sensitivity 89.50%, specificity 73.77%). For regional localization of positive LLNs, the model attained 77.27% accuracy and 80% specificity, while its performance in predicting maximum short-axis diameter reached 75% accuracy and 62.50% specificity. These results, supported by the confusion matrix (**Figure 2B**) and regional/diameter comparisons (**Figures 2C, D**), indicate robust capability in LLN status assessment.

### Identified the independent prognostic factors via Cox regression analyses and Kaplan-Meier survival analysis

We conducted univariate and multivariate Cox regression analyses to identify prognostic factors for rectal cancer (**Table 2**). For DFS, independent prognostic factors included sex (HR: 0.56, 95% CI: 0.34–0.90, *p* = 0.018), DLLLN status (HR: 2.02, 95% CI: 1.31–3.13, *p* = 0.002), and mrTD (HR: 4.45, 95% CI: 2.99–6.65, *p* < 0.001). For OS, independent prognostic factors included mrTD status (HR: 4.16, 95% CI: 2.25–7.71, *p* < 0.001), DLLLN status (HR: 2.46, 95% CI: 1.29–4.72, *p* = 0.01), mrCRM status (HR: 2.23, 95% CI: 1.21–4.11, *p* = 0.01).

**Table 2:** Univariate and Multivariate analysis for validation cohort.

	Disease-free survival				Overall survival			
	Univariate analysis		Multivariate analysis		Univariate analysis		Multivariate analysis	
	HR (95%CI)	P	HR (95%CI)	P	HR (95%CI)	P	HR (95%CI)	P
Age	1.01 (0.99-1.03)	0.336			1.00 (0.97-1.02)	0.836		
Sex								
Male	Ref		Ref		Ref			
Female	0.60 (0.37-0.97)	0.04	0.56 (0.35-0.92)	0.02	0.62 (0.31-1.26)	0.19		
CEA level ^a^								
Normal	Ref		Ref		Ref		Ref	
Abnormal	1.99 (1.33-2.96)	0.001	1.44 (0.95-2.17)	0.09	2.29 (1.25-4.20)	0.008	1.58 (0.84-2.96)	0.16
Location								
Low	Ref				Ref			
Middle & High	0.91 (0.64-1.28)	0.613			0.96 (0.58-1.59)	0.88		
mrT stage								
T1-T3	Ref				Ref			
T4	1.28 (0.81-2.17)	0.260			1.65 (0.84-3.27)	0.149		
mrN stage								
N0-N1	Ref				Ref			
N2	1.40 (0.93-2.10)	0.105			1.42 (0.77-2.60)	0.261		
mrTD								
Negative	Ref		Ref		Ref		Ref	
Positive	4.595 (3.10-6.82)	<0.001	3.88 (2.26-6.68)	<0.001	5.34 (2.95-9.69)	<0.001	4.06 (1.80-9.16)	0.001
mrCRM								
Clear	Ref		Ref		Ref		Ref	
Involved	1.66 (1.01-2.47)	0.01	1.23 (0.79-1.89)	0.36	3.0 (1.65-5.44)	<0.001	2.30 (1.21-4.37)	0.01
mrEMVI status								
Negative	Ref		Ref		Ref		Ref	
Positive	2.87 (1.94-4.29)	<0.001	1.00 (0.57-1.76)	1.00	3.17 (1.75-5.73)	<0.001	0.86 (0.38-1.96)	0.72
DLLLN status								
Negative	Ref		Ref		Ref		Ref	
Positive	2.69 (1.75-4.14)	<0.001	2.18 (1.41-3.37)	<0.001	2.88 (1.52-5.44)	0.001	2.45 (1.27-4.71)	0.01

HR, hazard ratio; CI, confidence interval; mr, magnetic resonance; DL, deep learning; CEA, carcinoembryonic antigen; CRM, circumferential resection margin; EMVI, extramural vascular invasion; LLN, lateral lymph node; TRG, Tumor regression score. ^a^The normal values for CEA level range from 0 to 5 ng/m.

**Figure 3:**
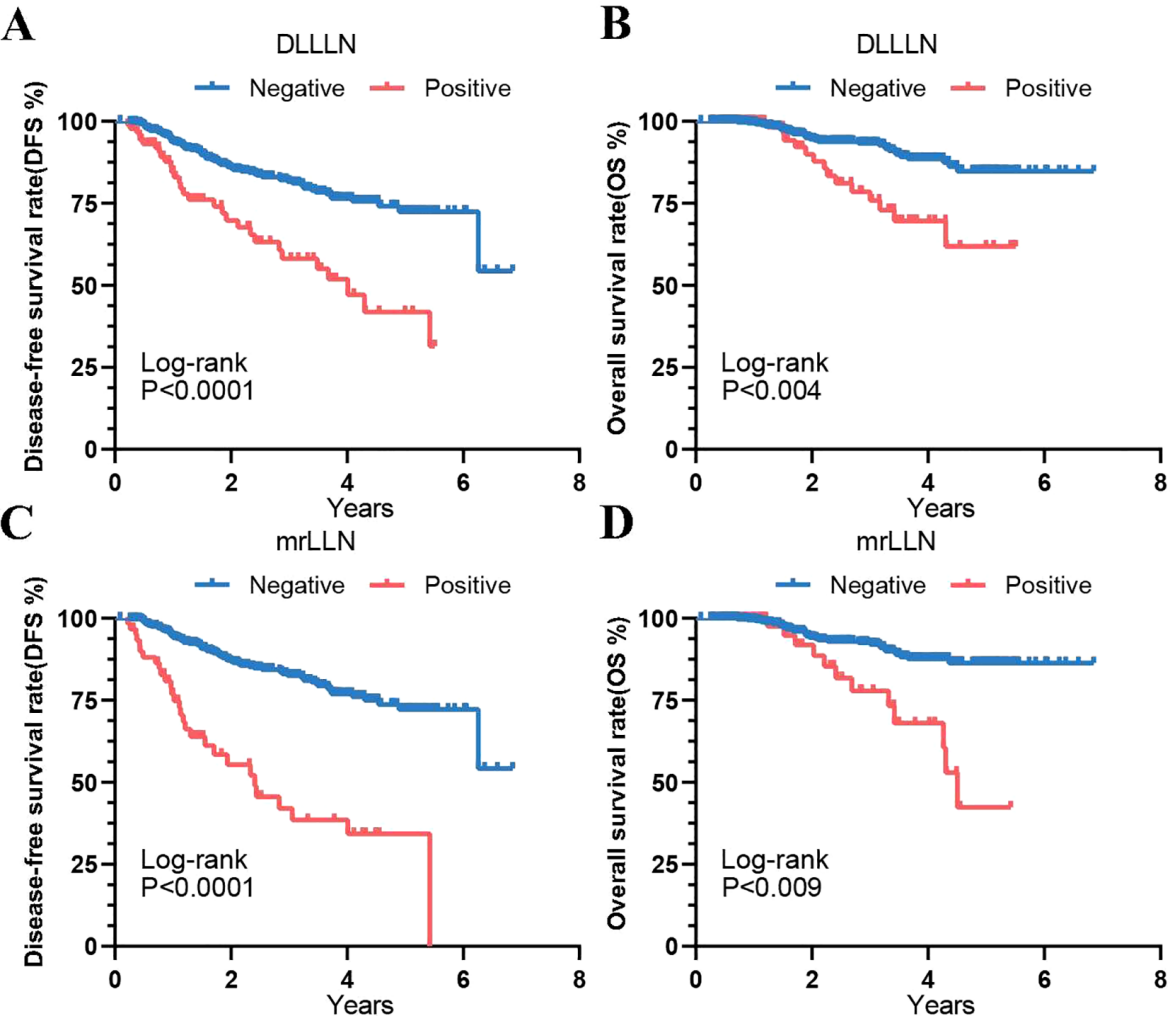
Kaplan-Meier curves showing prognostic outcomes of validation cohort patients predicted by the DL model and mrLLN. **(A)** Disease-Free Survival (DFS) predicted by the DL model. **(B)** Overall Survival (OS) predicted by the DL model. **(C)** DFS predicted by mrLLN. **(D)** OS predicted by mrLLN. Abbreviations: DL, deep learning; mr, magnetic resonance; LLN, lateral lymph node.

We subsequently performed Kaplan-Meier analysis to further assess the prognostic values of these factors. In the validation cohort, patients predicted by the DLLLN model with DLLLN+ demonstrated poorer prognoses (**Figures 3A, B**). Compared to DLLLN- patients, those with positive LLNs had lower DFS (HR = 2.668, 95% CI: 1.513-4.707, *p* = 0.0001) and OS (HR = 2.869, 95% CI: 1.224-6.723, *p* = 0.004). Additionally, survival curves were plotted based on unified assessments by the three radiologists (mrLLN) (**Figures 3C, D**). The result suggested that patients with mrLLN+ exhibited lower DFS (HR = 4.313, 95% CI: 2.149-8.655, *p* = 0.0001) and OS (HR = 3.472, 95% CI: 1.282-9.401, *p* = 0.009) than that of the patients with mrLLN-.

**Figure 4:**
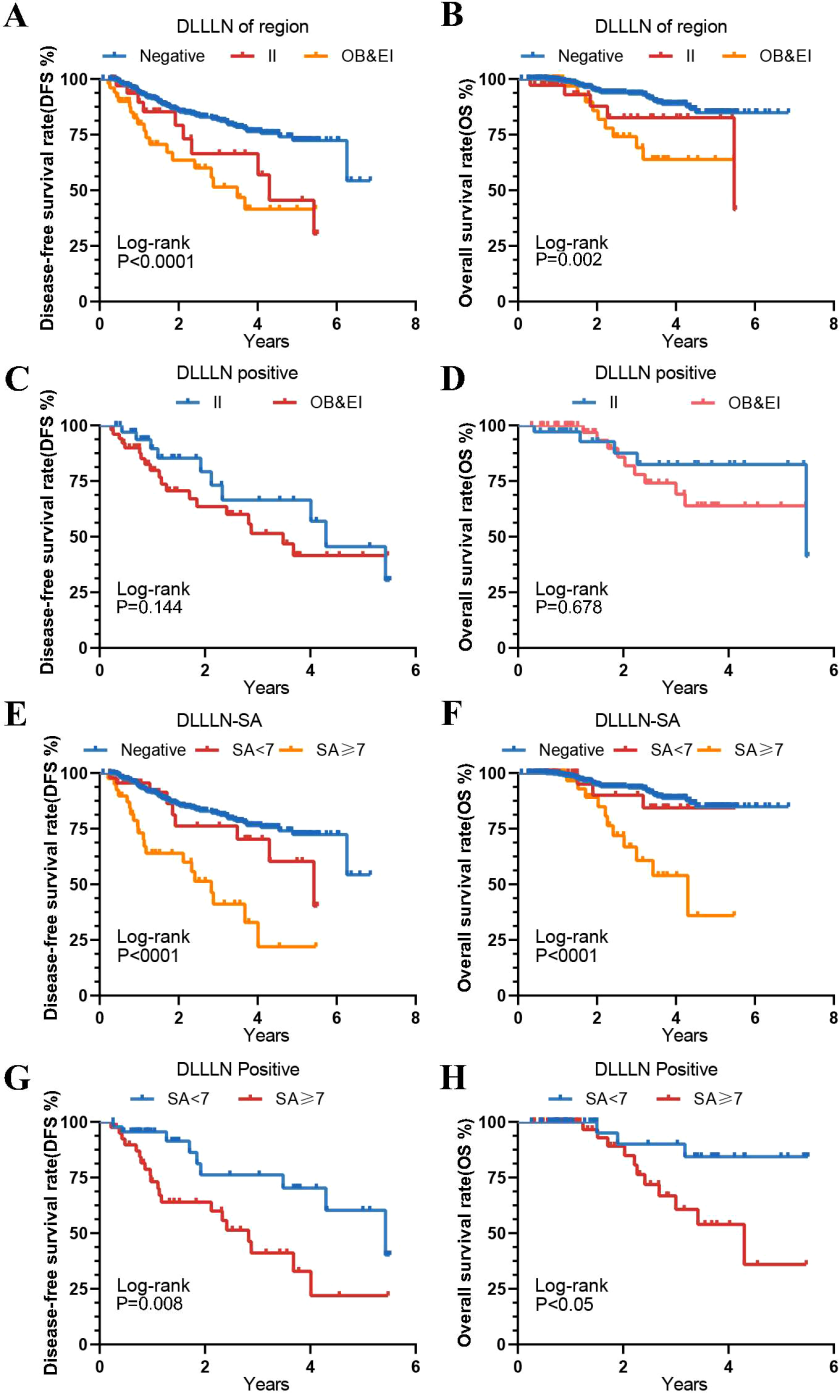
Kaplan-Meier curves for different DLLLN regions and different maximum short axis diameters. **(A, B)** Disease-Free Survival (DFS) and Overall Survival (OS) for patients with lateral lymph nodes (LLNs) in different regions, as predicted by the model. **(C, D)** DFS and OS among patients with positive DLLLN involvement across different anatomical regions (e.g., internal iliac [II] vs. obturator/external iliac [OB/EI]). **(E, F)** The DFS and OS for patients with different SA groups compared to those with positive DLLLN. **(G, H)** The DFS and OS for patients with positive LLNs predicted by the DL model, grouped by different SA categories.

We subsequently performed Kaplan-Meier analysis to further assess the survival outcomes of patients with positive lymph nodes in various regions. The results revealed no significant difference in DFS (HR = 1.539, 95% CI: 0.7401–3.200, *p* = 0.144) and OS (HR = 1.613, 95% CI: 0.5650-4.607, *p* = 0.768) between patients with positive lymph node metastases in the external iliac (EI) region and those in the obturator (OB) region (**Figures 4A–D**). Furthermore, for patients with different SA diameters, regardless of whether SA<7 mm or SA≥7 mm, those with positive lymph node metastases exhibited significantly lower and OS compared to the negative group (*p* < 0.001). Patients with a maximum lymph node SA diameter ≥7 mm had significantly worse DFS (HR = 2.815, 95% CI: 1.352-5.859, *p* = 0.008) and OS (HR = 3.9, 95% CI: 1.367-11.12, *p* = 0.05) than those with a diameter < 7 mm (**Figures 4E-H**).

Stratified analyses within the same mrN stage, based on DLLLN status (**Supplementary Figures 2A–F**), demonstrated that patients with positive LLNs exhibited significantly worse survival outcomes compared to those with negative LLNs, irrespective of the specific mrN stage. This finding underscores the role of positive LLNs as a robust and independent prognostic indicator of adverse outcomes. Furthermore, patients concurrently presenting with mrTD+ and DLLLN+ exhibited a marked deterioration in both DFS (HR = 9.844, 95% CI: 2.923-33.15, *p* < 0.0001) and OS (HR = 10.76, 95% CI: 1.594-72.59, *p* < 0.0001) when compared to patients who were negative for both factors (**Supplementary Figures 3A–F**). This highlights the synergistic prognostic impact of mrTD and positive LLNs on patient survival. In stratified analyses based on mrCRM status (**Supplementary Figures 4A–F**), patients with cooccurring mrCRM+ and DLLLN+ demonstrated significantly inferior DFS (HR = 6.092, 95% CI: 2.436-15.24, *p* < 0.0001) and OS (HR = 10.95, 95%CI: 2.975-40.30, *p* < 0.0001). Consistently, in stratified analyses by mrEMVI status, patients with concurrent mrEMVI+ and DLLLN+ exhibited markedly worse outcomes: DFS was reduced by a factor of 6.28 (95% CI 2.174–18.14, p < 0.001); OS was reduced by a factor of 7.34 (95% CI 1.443–37.30, p = 0.016) (**Supplementary Figures 5A–F**). Collectively, these findings highlight the prognostic significance of DLLLN+ when combined with either mrTD+, mrEMVI+, or mrCRM+ demonstrating its clinical utility as a robust predictor of patient outcomes.

## Discussion

LLNs are significant independent prognostic factor in low-lying rectal cancer. Studies show that patients with positive LLNs have a 5-year survival rate of only 30%, compared to 60%-80% for those without positive LLNs. Additionally, the presence of positive LLNs can increase the risk of postoperative local recurrence by 2–3 times (14, 25–29). Thus, preoperative assessment of positive LLNs is crucial for guiding treatment strategies and improving patient outcomes. In the present study, we developed an AI model, which demonstrated high efficacy in predicting positive LLN, providing an automated and efficient alternative to traditional radiological methods. Moreover, Cox regression analysis further confirmed that DL model-based prediction of positive LLN is an independent prognostic factor for rectal cancer. Compared to traditional methods that require manual ROI and feature extraction segmentation, DL models, which automatically segment ROIs and extract features, are more time-efficient and effective.

This study used a 7 mm cutoff for the maximum short-axis diameter of LLNs identified by the model. The model showed robust performance in predicting positive LLN, achieving 87.50% accuracy, 89.50% sensitivity, 73.77% specificity, a 26.23% false positive rate, and a 10.50% false negative rate. Prognostic analysis of the validation cohort showed that patients with positive LLNs had significantly shorter 3-year DFS (79.10% vs. 96.48%) and 5-year OS (61.62% vs. 84.82%) compared to those with negative LLNs. Several deep learning (DL) and radiomics studies have explored image-based prediction of lymph node metastasis in rectal cancer. For instance, Zhao et al. (30) developed an MRI-based radiomics model that achieved an AUC of 0.843 in the test cohort, while Yang et al. (31) reported a combined CT−MRI model with AUCs up to 0.957. However, such studies often face limitations in generalizability due to modest sample sizes and reliance on manual feature engineering, which introduces observer variability and increases overfitting risks. In contrast to these feature engineering-dependent methods, our approach employs an end-to-end deep learning framework that automatically learns discriminative features directly from raw imaging data. To leverage this advantage, our study developed and validated this fully automated framework on a large, multicenter cohort. It eliminates manual feature extraction, improves reproducibility, and enhances robustness—offering a more scalable and consistent solution for preoperative LLN assessment.

This study is the largest multicenter investigation to date, incorporating data from 1,000 patients across three centers for model training and an additional 480 patients from two independent hospitals for external validation. This comprehensive dataset significantly enhances the stability and generalizability of our predictive model. In addition, unlike traditional radiomics approaches that require manual delineation of regions of interest (ROIs) and feature extraction, our DL model automates these processes, improving efficiency and reducing time investment. Training on large-scale multicenter data, our DL model demonstrates superior predictive performance and clinical applicability. By leveraging extensive multicenter validation, our study refines model-building strategies, greatly improving prediction accuracy and clinical utility. In conclusion, this study overcomes the limitations of prior research by combining large-scale multicenter data with advanced DL techniques. This approach not only enhances the precision of positive lymph nodes prediction but also provides a solid foundation for optimizing treatment strategies and promoting personalized care in rectal cancer management.

To detect the independent prognostic factors for rectal cancer patients before surgery, we performed univariate and multivariate Cox regression analyses. The result suggested that mrTD, mrCRM, pCR status, and DLLLN status were significantly correlated with the prognosis of patients with rectal cancer. Further, we found that in all stratified analyses, we observed that patients with DLLLN+ had a significantly increased risk of mortality (DFS, OS) and a poorer prognosis. This result suggests that positive lymph LLN status is an independent prognostic factor for rectal cancer patients. DLLLN+ could serve as a prognostic marker to identify high-risk patients, thereby aiding in the development of individualized treatment plans that may enhance patient survival.

This study has several limitations. First, the reference standard was based on radiologic consensus rather than histopathology from lateral lymph node dissection, which may influence the accuracy of metastasis identification. Second, the model’s moderate specificity (73.8%) and considerable false-positive rate (26.2%) highlight the need for further refinement to reduce incorrect classifications. Third, although external validation involved centers from distinct geographic regions, broader multicenter studies are needed to strengthen generalizability. Finally, the model was trained only on T2‐weighted images; future integration of multiparametric MRI data could improve diagnostic precision.

## Conclusion

This multicenter study establishes a fully automated deep learning model for lateral lymph node assessment in rectal cancer, demonstrating both diagnostic accuracy and prognostic value. The model achieves robust performance in predicting LLN metastasis and serves as an independent prognostic factor for survival outcomes. By providing standardized, reproducible LLN evaluation, this approach enables reliable risk stratification and supports personalized treatment planning. Our findings position this DL model as a clinically viable tool to enhance preoperative decision-making and advance precision oncology in rectal cancer management.

## Data Availability

The raw data supporting the conclusions of this article will be made available by the authors, without undue reservation.
