# Supplementary material for: Deep learning based on MRI for assessing the prognostic value of lateral lymph nodes in rectal cancer
Source: Front Oncol. 2025 Nov 11;15:1681939. doi: 10.3389/fonc.2025.1681939 (PMC12643854; doi:10.3389/fonc.2025.1681939)
Supplement: Supplementary file 1 [file DataSheet1.docx]

**Supplementary Materials**

**Method S1 Inclusion and exclusion criteria**

For the recruitment of patients in the training cohort, we applied the following inclusion criteria: (1) Patients aged ≥18 years with a confirmed pathological diagnosis of rectal cancer; (2) Patients underwent pelvic MRI within two weeks prior to receiving neoadjuvant chemoradiotherapy (nCRT) or total mesorectal excision (TME), and had complete T2WI images. The exclusion criteria were as follows: (1) Lack of a confirmed pathological diagnosis; (2) Poor image quality; (3) Absence of pre-treatment pelvic MRI T2WI images.

The recruitment process for the two independent validation cohorts was notably more detailed and stringent (Supplementary Figure S1). The specific inclusion criteria were as follows: (1) Patients aged ≥18 years with a confirmed pathological diagnosis of rectal cancer; (2) Patients underwent standard total mesorectal excision (TME) within 8–12 weeks after receiving neoadjuvant chemoradiotherapy (nCRT); (3) Patients underwent pelvic MRI within two weeks prior to nCRT, with complete T2WI scans available; (4) Complete clinical information and pathological diagnosis results were available. The exclusion criteria were: (1) Presence of other malignancies or colorectal inflammatory history; (2) Patients who had undergone cancer treatment prior to the consultation; (3) Inability to obtain pathological response evaluation of the surgical specimen; (4) Incomplete or poor-quality MRI scans of LLN; (5) Missing or incomplete clinical and pathological information

**Method S2 Clinical information and the evaluation criteria for MRI T2WI indicators**

The clinical information was sourced from patients' medical records. Rectal cancer locations are categorized according to the tumor's distance from the dentate line: tumors located 0-5 cm away are considered low rectal cancers, those 5-10 cm are classified as mid rectal cancers, and tumors more than 10 cm from the dentate line are deemed high rectal cancers ^[1]^. The normal range for CEA levels is 0-5 ng/ml. TNM staging follows the guidelines outlined in the 9th edition of the TNM classification system ^[2]^.

**Reference:**

1. Valadão, M., Cesar, D., Véo, C. A. R., Araújo, R. O., do Espirito Santo, G. F., Oliveira de Souza, R., Aguiar, S., Jr, Ribeiro, R., de Castro Ribeiro, H. S., de Souza Fernandes, P. H., & Oliveira, A. F. (2022). Brazilian society of surgical oncology: Guidelines for the surgical treatment of mid-low rectal cancer. *Journal of surgical oncology*, *125*(2), 194–216. <https://doi.org/10.1002/jso.26676>
2. Janczewski, L. M., Faski, J., Nelson, H., Gollub, M. J., Eng, C., Brierley, J. D., Palefsky, J. M., Goldberg, R. M., Washington, M. K., Asare, E. A., Goodman, K. A., & American Joint Committee on Cancer Expert Panel on Cancers of the Lower Gastrointestinal, Anus Disease Site (2023). Survival outcomes used to generate version 9 American Joint Committee on Cancer staging system for anal cancer. *CA: a cancer journal for clinicians*, *73*(5), 516–523. https://doi.org/10.3322/caac.21780


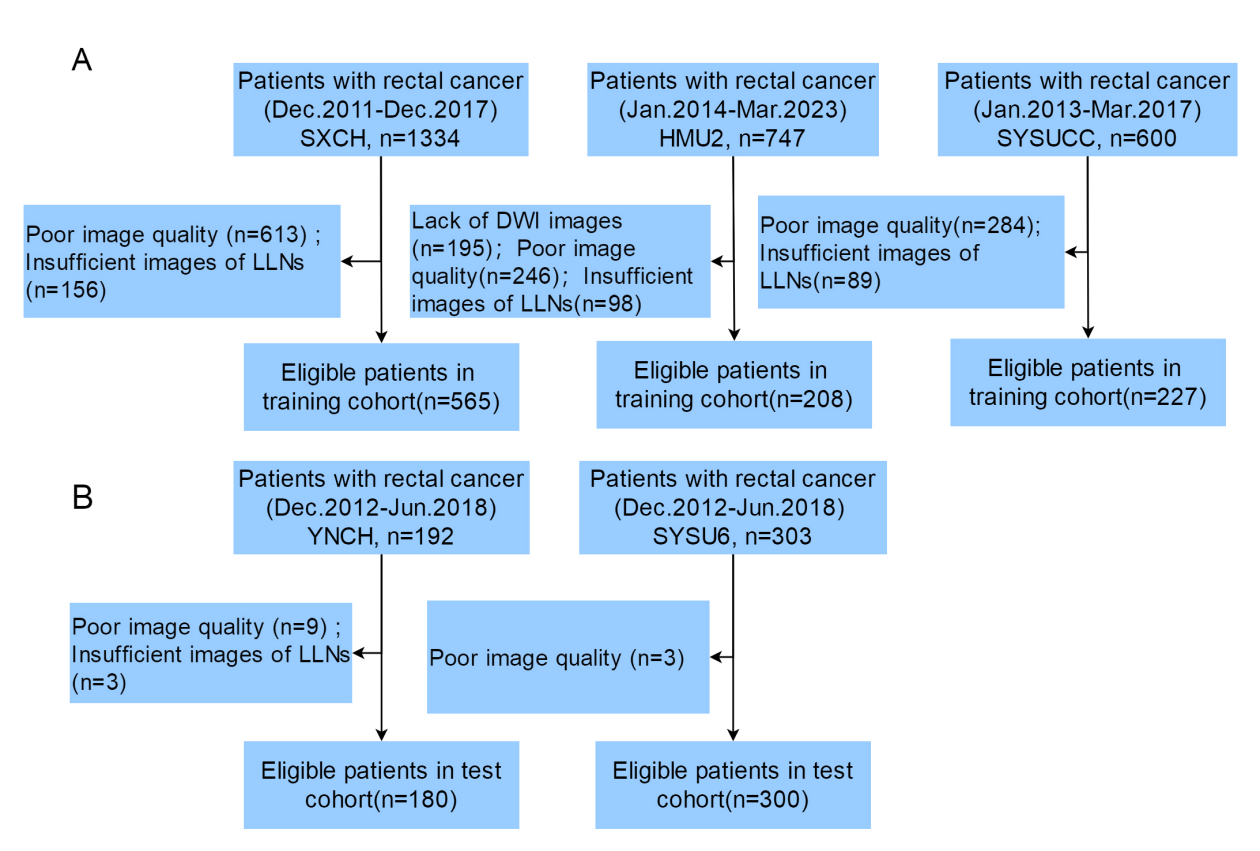


**Figure S1. The selection process of patients in the training and validation cohorts.** In the training cohort, patients were included from five independent centers (SXCH, Shanxi Cancer Hospital; SYSUCC, Sun Yat-sen University Cancer Center; HMU2, the Second Affiliated Hospital of Harbin Medical University). Patients in the validation cohort were from two independent centers (SYSU6, the Sixth Affiliated Hospital of Sun Yat-sen University; YNCH, Yunnan Cancer Hospital).

**
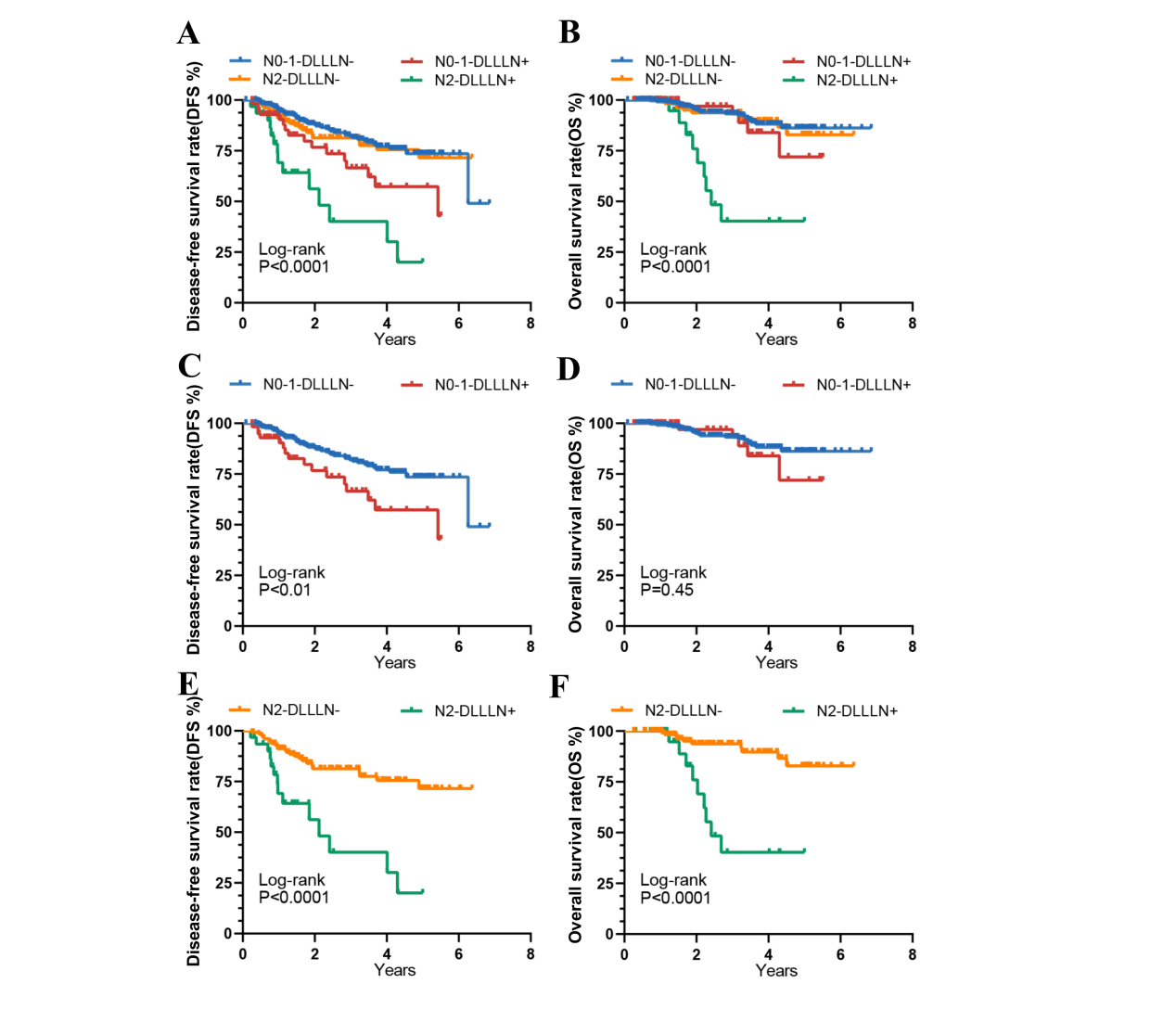
Figure S2. Kaplan-Meier curves for different mrN stages and DLLLN statuses.**

(A, B) DFS and OS for patients with different mrN stage and DLLLN status. (C, D) DFS and OS for mrN0-N1. (E, F) DFS and OS for mrN2.

**
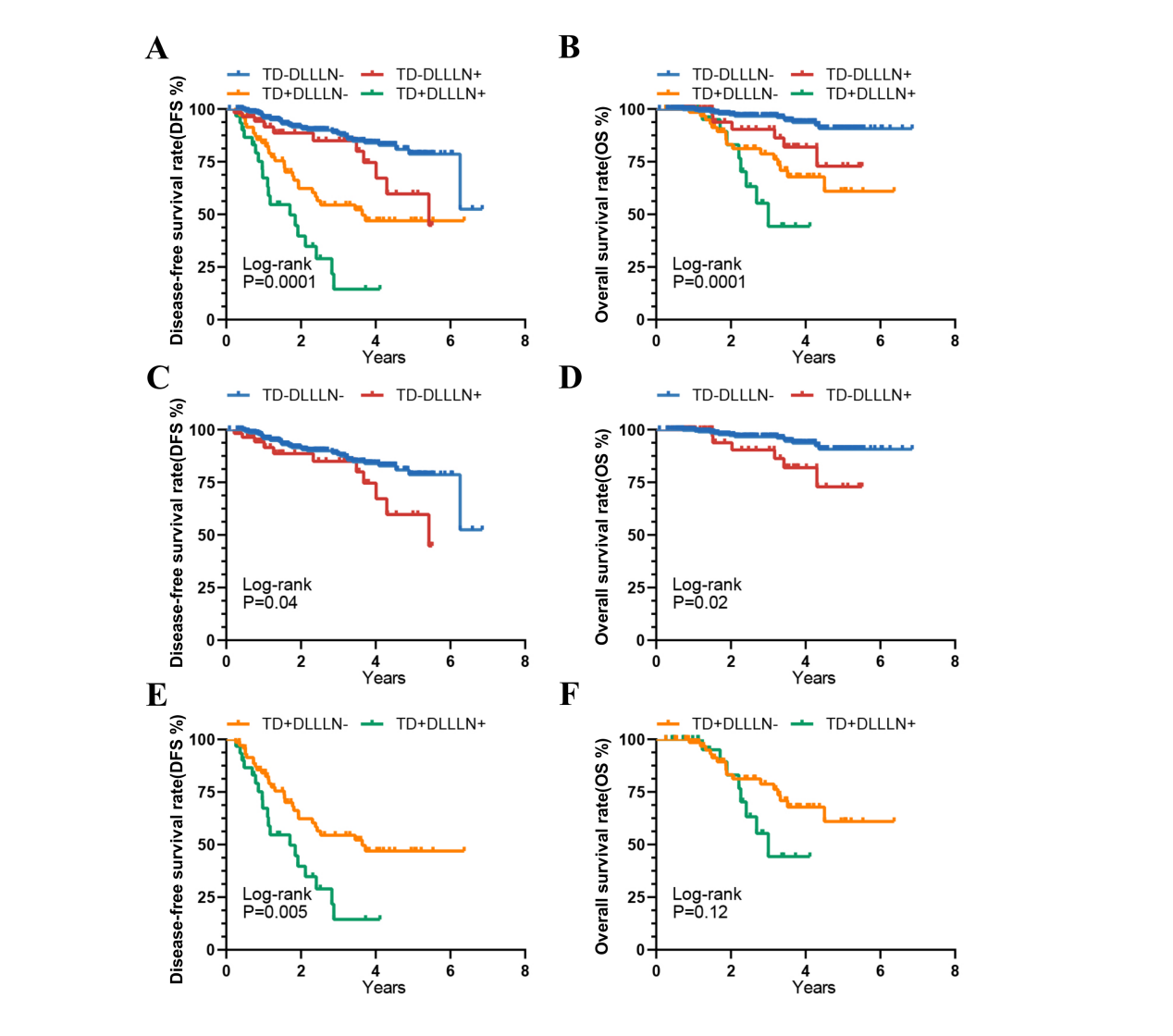
Figure S3. Kaplan-Meier curves for different** **mrTD and DLLLN statuses.**

(A, B) DFS and OS curves for patients with different mrTD and DLLLN status. (C, D) DFS and OS for different DLLLN statuses in patients with negative mrTD. (E, F) DFS and OS for different DLLLN statuses in patients with positive mrTD.

**
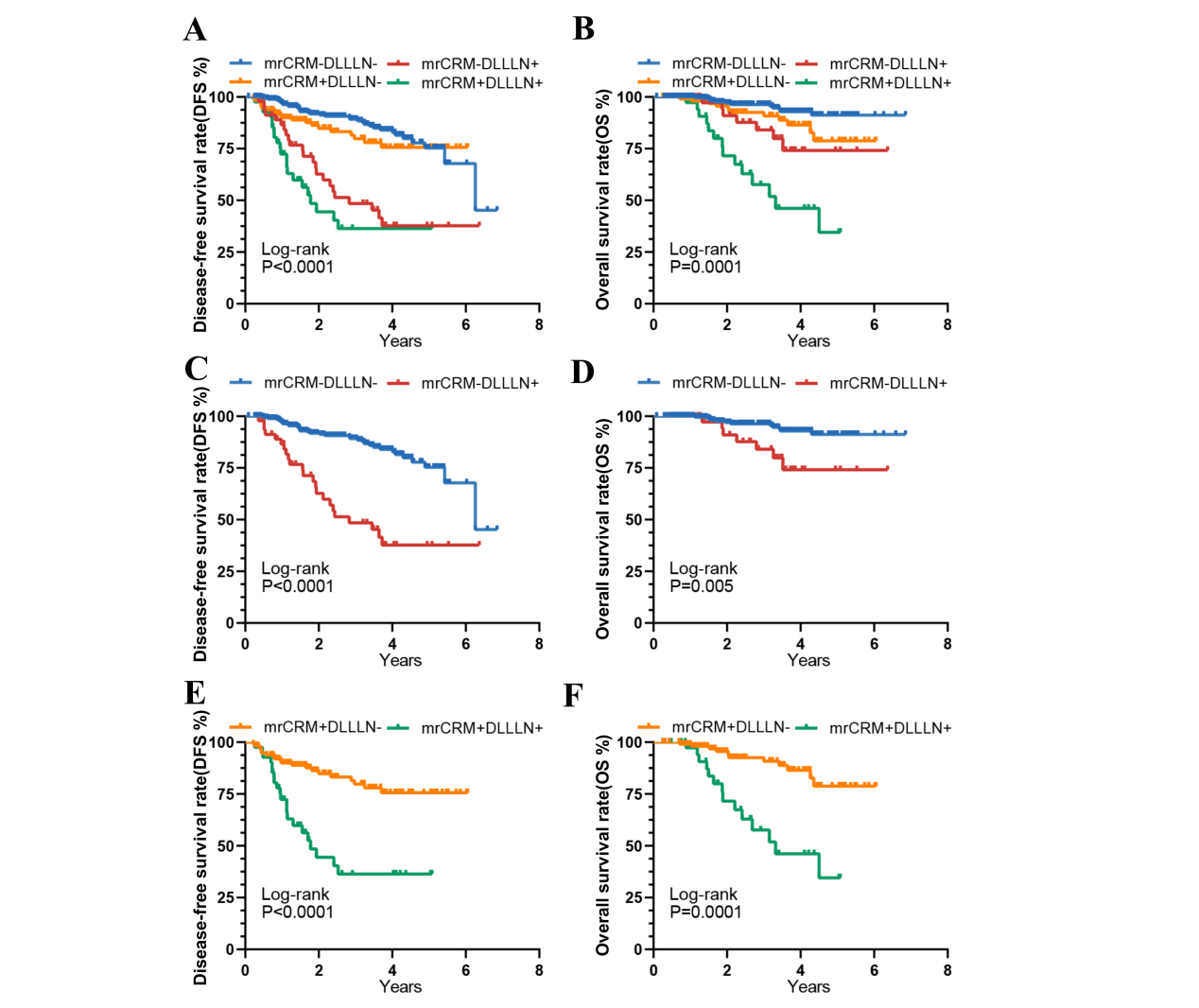
Figure S4. Kaplan-Meier curves for different** **mrCRM and DLLLN statuses.**

(A, B) DFS and OS curves for patients with different mrCRM and DLLLN status. (C, D) DFS and OS for different DLLLN statuses in patients with non-mrCRM. (E, F) DFS and OS for different DLLLN statuses in patients with mrCRM.


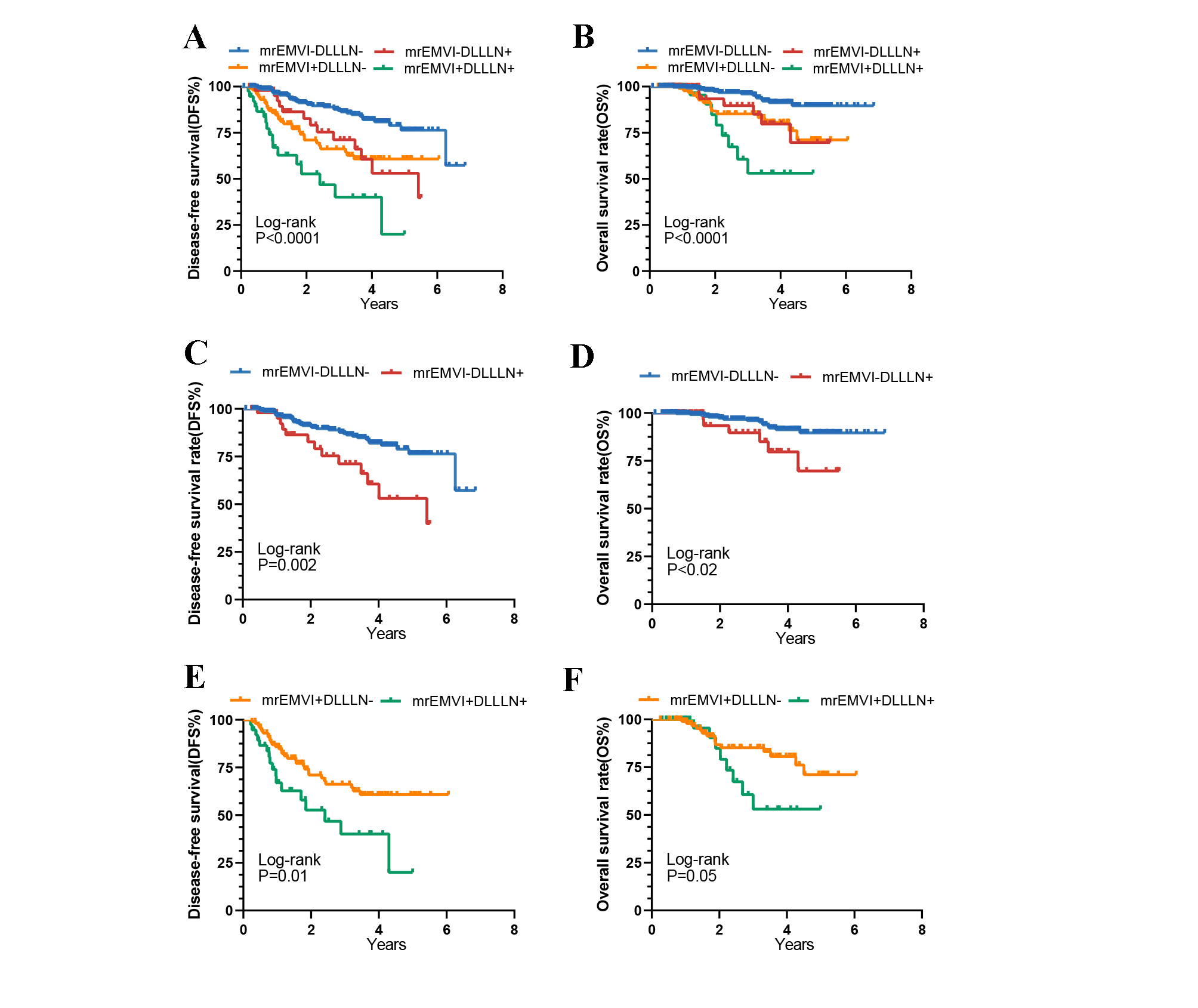
**Figure S5. Kaplan-Meier curves for different mrEMVI and DLLLN statuses.**

(A, B) DFS and OS curves for patients with different mrEMVI and DLLLN status. (C, D) DFS and OS for different DLLLN statuses in patients with non-mrEMVI. (E, F) DFS and OS for different DLLLN statuses in patients with mrEMVI.

**Table S1.** The MRI image acquisition parameters of the multiple centers

| **Hospital** | **Scanner** | **Sequence** | **TR/TE*(ms)** | **FOV(mm)** | **Matrix** | **Slice Thickness(mm)** | **Slice Gap(mm)** | **Flip Angle** |
| --- | --- | --- | --- | --- | --- | --- | --- | --- |
| **The Sixth Affiliated Hospital of Sun Yat- sen University** | GE3.0T (OPTIMA) | T2WI axial | 4300/104 | 100 | 288x256 | 3 | 6 | 90° |
|  |  | T2WI sagittal | 3578/104 | 100 | 288x256 | 3 | 3.5 | 90° |
|  |  | T2WI coronal | 3782/105.6 | 100 | 288x256 | 3 | 3.5 | 90° |
| **Sun Yat-sen University Cancer Center** | Philips 3.0T (Achieva) | T2WI axial | 2852/90 | 100 | 516x510 | 3 | 4 | 90° |
|  |  | T2WI sagittal | 3416/90 | 100 | 242x139 | 3 | 4 | 90° |
|  |  | T2WI coronal | 2852/100 | 100 | 387x222 | 5 | 6 | 90° |
|  | GE3.0T (DISCOVERY) | T2WI axial | 6480/81 | 100 | 382x384 | 4 | 5 | 90° |
|  |  | T2WI sagittal | 3883/90 | 100 | 436x218 | 3 | 4 | 90° |
|  |  | T2WI coronal | 3916/82 | 100 | 512x512 | 3 | 4 | 90° |
|  | GE 1.5T (SIGNA) | T2WI axial | 6000/92 | 60 | 320x224 | 5 | 6 | 90° |
|  |  | T2WI sagittal | 5903/91 | 100 | 512x512 | 5 | 6 | 90° |
|  |  | T2WI coronal | 5916/104 | 100 | 343x471 | 3 | 4 | 90° |
|  | SIEMENS 3.0T (Trio Tim) | T2WI axial | 3000/84 | 75 | 300x230 | 4 | 6 | 90° |
|  |  | T2WI sagittal | 2300/83 | 100 | 384x384 | 3 | 3.6 | 90° |
|  |  | T2WI coronal | 4400/81 | 100 | 320x320 | 6 | 7.2 | 90° |
| **Shanxi Cancer Hospital(train cohort)** | Philips 3.0T (Achieva) | T2WI axial | 3000/80 | 100 | 300x223 | 3 | 3.3 | 90° |
|  |  | T2WI sagittal | 4382.2/70 | 100 | 300x267 | 4 | 5 | 90° |
|  |  | T2WI coronal | 3000/80 | 100 | 300x218 | 3 | 4 | 90° |
| **Yunnan Cancer Hospital** | SIEMENS 1.5T (Avanto) | T2WI axial | 3200/100 | 100 | 288x320 | 3-4.5 | 3.3-5.2 | 90° |
|  | Philips 3.0T (Achieva) | T2WI axial | 4000/100 | 100 | 432x432 | 5 | 3.3-6 | 90° |
| **The Second Affiliated Hospital of Harbin Medical Hospital** | GE3.0T (OPTIMA) | T2WI axial | 4300/104 | 100 | 250x250 | 5 | 6 | 90° |
|  | GE 1.5T(SIGNA) | T2WI axial | 6000/92 | 100 | 250x250 | 5 | 6 | 90° |

**Abbreviation:** T2WI, T2-weighted images; TR, Repetition Time; TE, Echo Time; FOV, Field of view
